# Short‐ and Long‐Term Outcomes of Endoscopic Submucosal Dissection for Gastric Lesions in Elderly Patients Aged 80 Years or Older: Focus on Non‐Procedure‐Related Adverse Events

**DOI:** 10.1002/deo2.70334

**Published:** 2026-04-19

**Authors:** Hideharu Ogiyama, Yoko Murayama, Takuya Iihara, Harune Okuno, Kenji Hanafusa, Toshio Nakayama, Hiroko Fukushima, Hiromi Shimakoshi, Takahiro Amano, Hirotsugu Saiki, Nobuyasu Fukutake, Kunimaro Furuta, Sachiyo Kogita, Hisashi Ishida, Shusaku Tsutsui, Hiroyasu Iishi, Masahide Oshita

**Affiliations:** ^1^ Department of Gastroenterology and Hepatology Ikeda Municipal Hospital Ikeda Japan; ^2^ Department of Gastroenterology and Hepatology Itami City Hospital Itami Japan

**Keywords:** 80 and over, aged, endoscopic submucosal dissection, postoperative complications, prognosis, stomach neoplasms

## Abstract

**Objectives:**

With an aging society, the proportion of elderly patients undergoing gastric endoscopic submucosal dissection (ESD) is increasing. Although procedure‐related adverse events (AEs) are comparable between the elderly and non‐elderly patients, data on non‐procedure‐related AEs remain limited. This study aimed to evaluate the short‐ and long‐term outcomes of gastric ESD in elderly patients, focusing on risk factors for non‐procedure‐related AEs.

**Methods:**

We retrospectively analyzed 941 patients who underwent gastric ESD at two institutions between 2011 and 2024. Patients were stratified into elderly (≥80 years) and non‐elderly (<80 years) groups, and short‐ and long‐term outcomes were compared. Procedure‐related AEs included delayed bleeding, perforation, and stricture, whereas non‐procedure‐related AEs included pneumonia and other systemic complications.

**Results:**

Procedure‐related AE rates were similar between the groups (8.9% vs. 7.1%, *p* = 0.322), whereas the overall (13.8% vs. 8.3%, *p* = 0.010) and non‐procedure‐related AE rates (5.8% vs. 1.4%, *p* < 0.001) were significantly higher in elderly patients. Univariate analysis showed that Eastern Cooperative Oncology Group performance status (PS) ≥ 2 and Charlson Comorbidity Index (CCI) ≥ 2 were significant risk factors for non‐procedure‐related AEs among elderly patients. Non‐procedure‐related AEs were significantly associated with poorer overall survival (OS) (*p* = 0.013). Multivariate analysis identified systemic factors, including age, albumin level, CCI, and PS, as independent prognostic factors for OS in elderly patients.

**Conclusions:**

Assessment of systemic health status, particularly using PS and CCI, is essential for predicting non‐procedure‐related AEs, and they are useful indices for determining treatment indications in elderly patients undergoing gastric ESD.

**Trial Registration**: Not applicable.

## 1 Introduction

Endoscopic submucosal dissection (ESD) enables the en bloc resection of gastric neoplasms, thus allowing precise histopathological assessment while offering a minimally invasive treatment option. Advances have established gastric ESD as the standard therapeutic modality. However, the risk of adverse events (AEs) and appropriate indications for ESD in elderly patients have not been fully addressed.

Due to the aging population, gastric ESD is increasingly performed in elderly patients with comorbidities. The occurrence of AEs may lead to more severe outcomes, considering that comorbidities are more prevalent in older patients. Thus, careful monitoring and management of these patients are required.

The incidence rates of frequently occurring procedure‐related AEs such as delayed bleeding and perforation in elderly patients were comparable to those in non‐elderly individuals [1, 2, 3, 4]. Although several studies have described pneumonia as an additional AE, reports focusing on non‐procedure‐related AEs remain limited [2, 4, 5].

Thus, this study aimed to compare short‐term outcomes, including detailed analyses of non‐procedure‐related AEs, and long‐term outcomes between patients aged <80 years and those aged ≥80 years undergoing gastric ESD.

## 2 Methods

### 2.1 Patients and Study Design

This retrospective study was conducted in two tertiary hospitals. We enrolled 941 consecutive patients with 1010 gastric lesions who underwent gastric ESD for early gastric cancer (EGC) or suspected gastric neoplastic lesions between April 2011 and December 2024.

**FIGURE 1:**
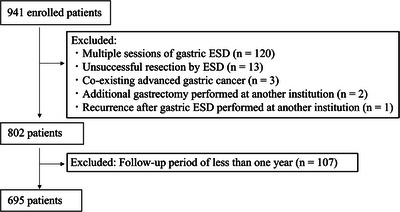
Flowchart of patient selection for the analysis of long‐term prognosis.

After applying the exclusion criteria shown in Figure 1, 695 patients were included in the long‐term prognostic analysis. The study population was stratified into patients aged ≥80 years (elderly patients) and those aged <80 years (non‐elderly patients).

### 2.2 ESD Procedure

Gastric ESD was performed using standard techniques. After the lesion was resected, preventive coagulation of visible vessels in the resection area was performed using hemostatic forceps. Operators were classified as trainees (<30 prior ESDs) or experienced endoscopists (≥30); all trainees were supervised by experienced endoscopists.

Sedation was achieved using midazolam (Dormicum; Astellas Pharma, Tokyo, Japan) and pethidine hydrochloride (Takeda Pharma, Osaka, Japan), with additional doses administered as needed to maintain a Modified Observer's Assessment of Alertness/Sedation score of 3 [6].

### 2.3 Management of Anticoagulant and Antiplatelet Agents

Perioperative anticoagulant administration was performed according to the guidelines of the Japanese Gastroenterological Endoscopy Society [7, 8], and the duration of antithrombotic drug interruption was minimized.

### 2.4 Data Collection

Data on patient characteristics (age, sex, use of anticoagulants and antiplatelet agents, comorbidities, Eastern Cooperative Oncology Group performance status [PS], and body mass index [BMI]) and laboratory parameters were collected. The Charlson Comorbidity Index (CCI) was calculated based on documented comorbidities [9]. The estimated glomerular filtration rate (eGFR) was calculated using the Japanese equation [10].

Data on lesion location, macroscopic type and size, estimated invasion depth, and ulcerative findings were collected. For patients who underwent multiple same‐day resections, only the lesions with the highest histological grade (or, if tied, the largest) were included. For therapeutic outcomes, we collected data on the procedure time, specimen and lesion size, en bloc resection rate, postoperative histopathology, and curability, which were evaluated using a scoring system [11]. In non‐curative cases, eCuraC‐2 status, indicating potential lymph node metastasis risk, was assessed based on histopathological findings. Delayed bleeding was defined as clinical evidence of bleeding that required endoscopic hemostasis or a decrease in hemoglobin level >2 g/dL after ESD. Perforation was diagnosed based on the presence of free air on computed tomography after ESD. A stricture was defined as a narrowing of the gastric lumen that prevented the passage of a standard 10‐mm diameter endoscope and necessitated endoscopic balloon dilation. Pneumonia was diagnosed on radiography or computed tomography, accompanied by a fever of 38°C or higher, rales, or cough.

AEs during the short‐term period (within 30 days of ESD) were categorized as procedure‐related (technical AEs: delayed bleeding, perforation, and stricture) or non‐procedure‐related (systemic AEs: pneumonia and other medical conditions that were not directly caused by the ESD procedure). Minor AEs were excluded, and only AEs that influenced the procedure or extended the hospital stay were documented. Procedure‐related mortality was defined as death within 30 days of ESD.

### 2.5 Measured Outcomes

Short‐term outcomes, including procedure‐related and non‐procedure‐related AEs, were defined as those within 30 days of the ESD procedure. The primary outcome was the rate of non‐procedure‐related AEs. Secondary outcomes included the overall AE rate, rate of procedure‐related AEs, and treatment outcomes such as en bloc resection rate and long‐term prognosis. In addition, patient backgrounds, lesion characteristics, therapeutic outcomes, and potential risk factors for AEs in elderly patients were evaluated. The details of both procedure‐ and non‐procedure‐related AEs were collected and analyzed. Furthermore, poor prognostic factors associated with overall survival (OS) were analyzed in elderly patients by assessing their baseline clinical characteristics and the impact of non‐procedure‐related AEs.

### 2.6 Ethics

This study was performed in accordance with the Declaration of Helsinki and the Ethical Guidelines for Medical and Health Research Involving Human Subjects of the Ministry of Health, Labor, and Welfare and the Ministry of Education, Culture, Sports, Science, and Technology in Japan. This study was approved by the ethics committees of Ikeda Municipal Hospital and Itami City Hospital. All patients were provided with the opportunity to decline participation in the study using the opt‐out method at each hospital. The requirement for informed consent was waived.

### 2.7 Statistical Analysis

Continuous and categorical variables are presented as means ± standard deviation and proportions, respectively. Data were compared using the Student's t‐test, chi‐square test, or Fisher's exact test, as appropriate. The cutoff value for the CCI was defined using receiver operating characteristic (ROC) analysis. Multivariate logistic regression analysis was performed to identify the risk factors that significantly affected AEs. Significant variables in univariate analysis were included in multivariate analysis. Survival curves were constructed using the Kaplan–Meier method and compared using the log‐rank test. The Cox proportional hazards model was used to assess the factors associated with prognosis. Differences were considered statistically significant at *p* < 0.05.

Statistical analyses were performed using the JMP software (ver. 12.2.0; SAS Institute Inc., Cary, NC, USA).

## 3 Results

Table 1 summarizes the baseline characteristics of the elderly and non‐elderly patients. In the ROC analysis, 2 was the optimal CCI cutoff (area under the curve 0.72; 95% confidence interval [CI]: 0.621–0.818; Figure S1). Elderly patients had a higher proportion of females and were more likely to have multiple comorbidities, leading to higher CCI and PS scores than non‐elderly patients. Elderly patients were also more likely to receive antiplatelet therapy and had lower levels of hemoglobin, albumin, and eGFR. Among the lesion characteristics, lesion size was significantly but only slightly larger in elderly patients.

**TABLE 1:** Comparison of baseline characteristics in elderly (≥80) and non‐elderly (<80) patients.

	Total (*n* = 941)	Elderly (≥80) (*n* = 291)	Non‐elderly (<80) (*n* = 650)	*p*‐value
Age (years, mean ± SD)	74.9 ± 8.4	83.6 ± 3.2	71.0 ± 7.0	<0.001
Sex (male), n (%)	680 (72.2)	197 (67.7)	483 (74.3)	0.036
BMI (kg/m^2^, mean ± SD)	23.1 ± 3.4	22.9 ± 3.3	23.2 ± 3.4	0.255
Hypertension, *n* (%)	474 (50.4)	170 (58.4)	304 (46.8)	0.001
Hyperlipidemia, *n* (%)	244 (25.9)	72 (24.7)	172 (26.5)	0.578
Hemodialysis, *n* (%)	10 (1.1)	3 (1.0)	7 (1.1)	1.000
Liver cirrhosis, *n* (%)	36 (3.8)	15 (5.2)	21 (3.2)	0.155
Ischemic heart disease, *n* (%)	120 (12.8)	57 (19.6)	63 (9.7)	<0.001
Chronic heart failure, *n* (%)	65 (6.9)	32 (11.0)	33 (5.1)	<0.001
Atrial fibrillation, *n* (%)	29 (3.1)	14 (4.8)	15 (2.3)	0.040
Diabetes, *n* (%)	179 (19.0)	56 (19.2)	123 (18.9)	0.908
Cerebrovascular disease, *n* (%)	43 (4.6)	20 (6.9)	23 (3.5)	0.024
Dementia, *n* (%)	24 (2.6)	15 (5.2)	9 (1.4)	<0.001
Metastatic neoplasm, *n* (%)	15 (1.6)	7 (2.4)	8 (1.2)	0.184
Antiplatelet therapy, *n* (%)	199 (21.1)	96 (33.0)	103 (15.9)	<0.001
Anticoagulant therapy, *n* (%)	80 (8.5)	32 (11.0)	48 (7.4)	0.066
Hemoglobin (g/dL, mean ± SD)	13.3 ± 1.7	12.6 ± 1.7	13.5 ± 1.6	<0.001
Albumin (g/dL, mean ± SD)	4.03 ± 0.41	3.88 ± 0.42	4.10 ± 0.39	<0.001
eGFR (mL/min/1.73 m^2^, mean ± SD)	63.3 ± 17.8	58.3 ± 17.7	65.5 ± 17.4	< 0.001
CCI ≥ 2, *n* (%)	199 (21.1)	85 (29.2)	114 (17.5)	<0.001
PS ≥ 2, *n* (%)	32 (3.4)	26 (8.9)	6 (0.9)	<0.001
Location (U/M/L/remnant), *n*	133/294/480/34	39/93/146/14	94/201/335/20	0.492
Macroscopic type (elevated/flat or depressed), *n*	501/440	162/129	339/311	0.318
Lesion size (mm, mean ± SD)	14.5 ± 10.1	15.9 ± 10.7	14.0 ± 9.8	0.012
Lesion depth (pre‐ESD) cT1b (SM), *n* (%)	30 (3.1)	13 (4.5)	17 (2.6)	0.135
Ulceration findings, *n* (%)	74 (7.9)	25 (8.6)	49 (7.5)	0.579

Abbreviations: BMI, body mass index; CCI, charlson comorbidity index; eGFR, estimated glomerular filtration rate; PS, eastern cooperative oncology group performance status; SD, standard deviation; SM, submucosa.

Table 2 presents the therapeutic outcomes and AEs in elderly and non‐elderly patients. No significant differences were observed between the two groups in terms of en bloc resection or procedure time. Histopathology confirmed gastric cancer in 84.2% of patients and non‐cancerous lesions in 15.8%. The proportion of patients with gastric cancer was slightly higher in the elderly patients than in non‐elderly patients (88.0% vs. 82.5%, *p* = 0.032). However, the curability rate did not differ significantly between the two groups. Although the frequency of procedure‐related AEs, including delayed bleeding, intraoperative perforation, and stricture, did not differ significantly between the groups (8.9% vs. 7.1%, *p* = 0.322), the overall AE rate was significantly higher in the elderly patients (13.8%) than in the non‐elderly patients (8.3%) (*p* = 0.010), primarily because of the higher incidence of non‐procedure‐related AEs, such as pneumonia (5.8% vs. 1.4%, *p* < 0.001). Multiple concurrent AEs were also more frequent in elderly patients (2.0% vs. 0.2%, *p* = 0.004). Procedure‐related mortality occurred in two elderly patients.

**TABLE 2:** Therapeutic outcomes and adverse events in elderly (≥80) and non‐elderly (<80) patients.

	Total (*n* = 941)	Elderly (≥80) (*n* = 291)	Non‐elderly (<80) (*n* = 650)	*p*‐value
Resected specimen size (mm, mean ± SD)	35.7 ± 13.0	36.2 ± 13.2	35.5 ± 12.9	0.451
Resected lesion size (mm, mean ± SD)	16.3 ± 12.1	17.0 ± 11.2	14.9 ± 11.0	0.006
En bloc resection rate, *n* (%)	900 (95.6)	278 (95.5)	622 (95.7)	0.912
Procedure time (min, mean ± SD)	79.2 ± 57.4	78.7 ± 59.5	79.5 ± 56.5	0.833
Operator experience (trainee)^a^, *n* (%)	387 (41.1)	120 (41.2)	267 (41.1)	0.963
Histopathology, *n* (%)				
Cancer	792 (84.2)	256 (88.0)	536 (82.5)	0.032
Adenoma	116 (12.3)	29 (10.0)	87 (13.4)	0.140
^b^Others	33 (3.5)	6 (2.1)	27 (4.2)	0.107
Ulcerative findings, *n* (%)	79 (8.4)	25 (8.6)	54 (8.3)	0.885
Curability^c^ eCuraC‐2/nonC‐2/non‐resected, n	94/833/14	37/249/5	57/584/9	0.157
Adverse events (AE), *n* (%)	94 (10.0)	40 (13.8)	54 (8.3)	0.010
^d^Procedure‐related AEs	72 (7.7)	26 (8.9)	46 (7.1)	0.322
Delayed bleeding, *n* (%)	54 (5.7)	20 (6.9)	34 (5.2)	0.317
Intraoperative perforation, *n* (%)	17 (1.8)	6 (2.1)	11 (1.7)	0.694
Stricture, *n* (%)	2 (0.2)	1 (0.3)	1 (0.2)	0.523
Non‐procedure‐related AEs, *n* (%)	26 (2.8)	17 (5.8)	9 (1.4)	<0.001
Pneumonia, *n* (%)	13 (1.4)	8 (2.7)	5 (0.8)	0.016
Multiple concurrent AEs, n (%)	7 (0.7)	6 (2.0)	1 (0.2)	0.004
Procedure‐related mortality	2 (0.2)	2 (0.7)	0 (0.0)	0.095
Hospital stay (day, mean ± SD)	8.6 ± 4.4	9.0 ± 4.4	8.4 ± 4.3	0.161

Abbreviation: SD, standard deviation.

^a^ Trainee endoscopists were defined as one who had performed ≤30 gastric ESD procedures.

^b^ Others include neuroendocrine tumors/carcinomas, MALT lymphomas, and hyperplastic polyps.

^c^ Curability was evaluated using a scoring system^11^. Non‐resected cases refer to lesions for which ESD could not be successfully completed. NonC‐2 lesions exclude eCuraC‐2 and non‐resected cases, including non‐cancerous lesions.

^d^ Procedure‐related AEs include delayed bleeding, intraoperative perforation, and strictures.

The various non‐procedure‐related AEs observed in elderly patients, including thromboembolic events (myocardial and cerebral infarctions) and other systemic complications, are shown in Table 3. Bradycardia was the only non‐procedure‐related AE observed.

**TABLE 3:** Summary of Common Terminology Criteria for Adverse Events (CTCAE) grades for adverse events affecting length of hospital stay, excluding delayed bleeding, perforation, and pneumonia.

	Grade 2	Grade 3	Grade 4	Total
Cholelithiasis, cholecystitis	2	1	0	3
Arrhythmia (atrial fibrillation, bradycardia)	0	2	0	2
Urinary tract infection	0	2	0	2
Delirium	0	2	0	2
Urinary retention	1	0	0	1
Hyponatremia	0	1	0	1
Myocardial infarction	0	1	0	1
Cerebral infarction	0	0	1	1
Fever of unknown origin	0	1	0	1
Hemorrhagic rectal ulcer	0	1	0	1

Abbreviations: CTCAE, common terminology criteria for adverse events.

Univariate analysis of factors associated with both all AEs and non‐procedure‐related AEs in elderly patients showed that CCI ≥ 2 and PS ≥ 2 were significantly associated with the occurrence of AEs (Table S1 and Table 4). Multivariate logistic regression analysis identified CCI ≥ 2 (odds ratio [OR] = 2.38, 95% CI = 1.16–4.83, *p* = 0.018) and PS ≥ 2 (OR = 3.87, 95% CI = 1.52–9.49, *p* = 0.005) as independent risk factors for overall AEs in elderly patients (Table S2). In an exploratory multivariate analysis for non‐procedure‐related AEs (due to the limited number of events, *n* = 17), PS ≥ 2 was also identified as a significant independent risk factor (OR = 3.94, *p* = 0.034) (Table 5).

**TABLE 4:** Univariate analysis of factors associated with non‐procedure‐related adverse events (AE) in elderly patients.

	Non‐procedure‐related AE (*n* = 17)	Without non‐procedure‐related AE (*n* = 274)	*p*‐value
Very elderly ≥85, *n* (%)	6 (35.3)	86 (31.4)	0.737
Sex (male), *n* (%)	13 (76.5)	184 (67.2)	0.425
Antiplatelet therapy, *n* (%)	6 (35.3)	90 (32.9)	0.835
Anticoagulant therapy, *n* (%)	3 (17.7)	29 (10.6)	0.744
Hemoglobin (g/dL, mean ± SD)	12.3 ± 1.8	12.6 ± 1.7	0.400
Albumin (g/dL, mean ± SD)	3.68 ± 0.66	3.89 ± 0.40	0.051
eGFR (mL/min/1.73 m^2^, mean ± SD)	55.9 ± 11.2	58.4 ± 18.0	0.563
BMI (kg/m^2^, mean ± SD)	22.2 ± 3.3	22.9 ± 3.3	0.410
CCI ≥ 2	9 (47.1)	77 (28.1)	0.029
PS ≥ 2	5 (29.4)	21 (7.7)	0.002
Location (U/M/L/remnant), *n*	2 / 5 / 9 / 1	37 / 88 / 136 / 13	0.985
Macroscopic type (elevated/flat or depressed), *n*	9 / 8	153 / 121	0.815
Lesion size (mm, mean ± SD)	17.1 ± 7.3	18.0 ± 12.0	0.838
Procedure time (min, mean ± SD)	86.9 ± 58.4	78.1 ± 59.7	0.559
Operator experience (trainee) ^a^, *n* (%)	8 (47.1)	112 (40.9)	0.615
Curability^b^ eCuraC‐2/nonC‐2/non‐resected, *n*	2 / 15 / 0	35 / 234 / 5	0.845

Abbreviations: BMI, body mass index; CCI, charlson comorbidity index; eGFR, estimated glomerular filtration rate; PS, eastern cooperative oncology group performance status; SD, standard deviation.

^a^ Trainee endoscopists were defined as one who had performed ≤30 gastric ESD procedures.

^b^ Curability was evaluated using a scoring system^11^. Non‐resected cases refer to lesions for which ESD could not be successfully completed. NonC‐2 lesions exclude eCuraC‐2 and non‐resected cases, including non‐cancerous lesions.

**TABLE 5:** Multivariate logistic regression analysis of factors associated with non‐procedure‐related adverse events in elderly patients.

	Odds ratio	95% (CI)	*p*‐value
CCI ≥ 2	2.30	0.81–6.54	0.117
PS ≥ 2	3.94	1.12–12.3	0.034

Abbreviations: CCI, charlson comorbidity index; CI, confidence interval; PS, eastern cooperative oncology group performance status.

**FIGURE 2:**
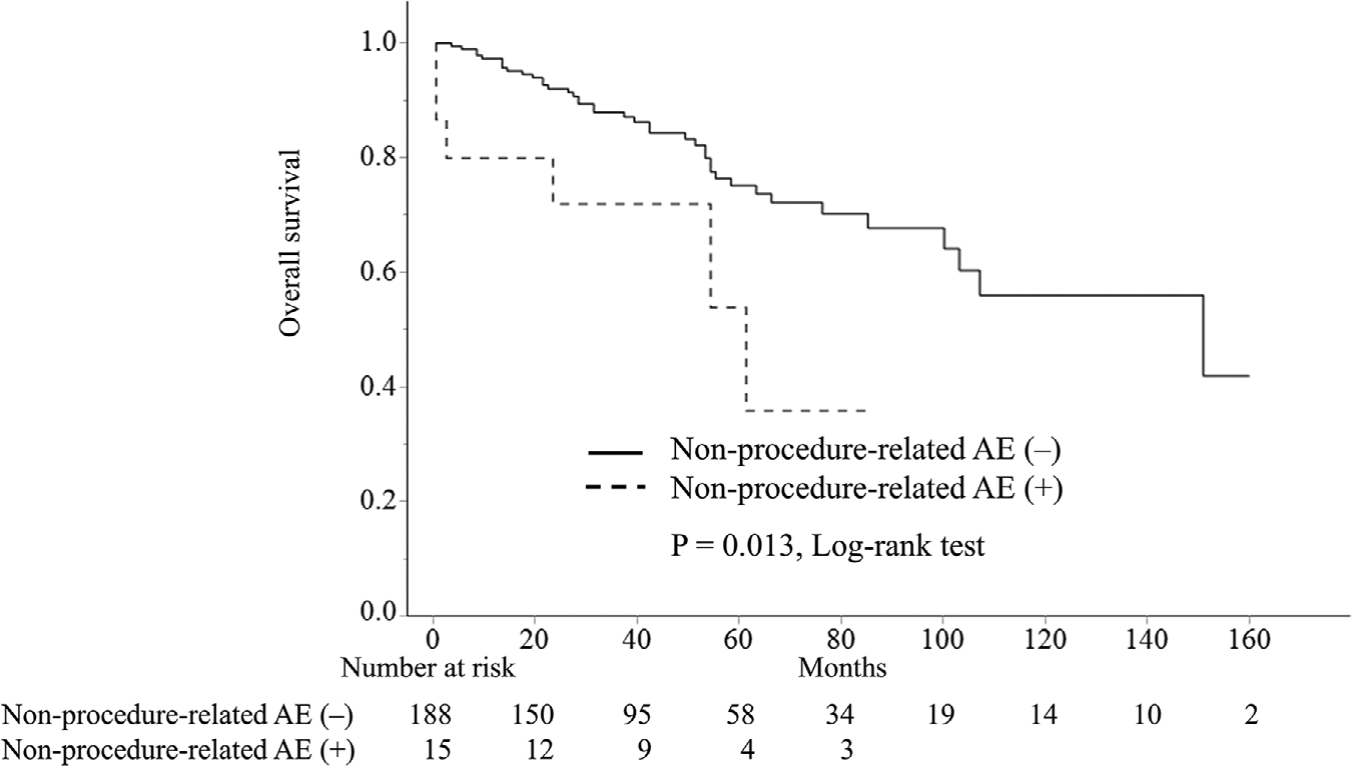
Kaplan–Meier curves showing overall survival in elderly patients with or without non‐procedure‐related adverse events (AEs). Overall survival was significantly poorer in elderly patients with non‐procedure‐related AEs (dashed line) than in those without AEs (solid line) (log‐rank test, *p* = 0.013). The number at risk for each time point is shown in the following graph.

Elderly patients had significantly poorer OS (*p* < 0.001; Figure S2). The median follow‐up period for the 695 patients was 64 months (range: 12–168 months). Patients with non‐procedure‐related AEs had significantly shorter OS (*p* = 0.013) (Figure 2). Six of 17 elderly patients with non‐procedure‐related AEs died (all non‐gastric cancer), including two AE‐related deaths. Table 6 presents the results of Cox proportional hazards analysis for long‐term prognosis in elderly patients. Advanced age, low albumin level, CCI ≥ 2, and PS ≥ 2 were identified as significant predictors of poor prognosis. During the observation period, 105 deaths occurred, with 2 deaths attributable to gastric cancer. One patient underwent additional surgery and was diagnosed with stage IB disease, but developed postoperative recurrence and eventually died of the disease. The other patient did not undergo additional surgery because of poor general condition and died of metastatic recurrence.

**TABLE 6:** Cox regression analysis for overall survival in elderly patients.

	Univariate analysis			Multivariate analysis		
	**Hazard ratio**	**95% (CI)**	***p*‐value**	**Hazard ratio**	**95% (CI)**	***p*‐value**
Age (years)	1.13	1.03–1.24	0.010	1.13	1.02–1.25	0.015
Sex Male	1.96	1.02–4.05	0.042	1.89	0.95–4.03	0.070
Hemoglobin (g/dL)	0.72	0.61–0.86	<0.001	0.84	0.68–1.04	0.101
Albumin (g/dL)	0.21	0.12–0.37	<0.001	0.28	0.13–0.59	0.001
eGFR (mL/min/1.73 m^2^)	0.99	0.97–1.01	0.325			
BMI (kg/m^2^)	0.95	0.86–1.03	0.221			
CCI ≥ 2	3.70	2.04–6.90	<0.001	2.23	1.14–4.42	0.019
PS ≥ 2	8.31	3.76–16.98	<0.001	8.60	3.55–20.02	<0.001
Cancer	1.36	0.77–2.71	0.308			
Curability^a^ eCuraC‐2	1.27	0.57–2.51	0.535			

Abbreviations: BMI, body mass index; CCI, charlson comorbidity index; CI, confidence interval; eGFR, estimated glomerular filtration rate; PS, eastern cooperative oncology group performance status.

^a^ Curability was evaluated using a scoring system^11^.

## 4 Discussion

We comprehensively analyzed the short‐ and long‐term outcomes of gastric ESD in elderly patients aged ≥80 years, with a particular focus on non‐procedure‐related AEs, which have rarely been reported. To the best of our knowledge, this is one of the first studies to categorize and evaluate non‐procedure‐related AEs, including systemic AEs beyond pneumonia, in elderly patients undergoing gastric ESD. The novelty of the current study lies in highlighting the significantly higher incidence of non‐procedure‐related AEs in elderly patients and that they are significantly associated with poorer OS. While non‐procedure‐related AEs significantly predicted poorer OS, most associated deaths were indirect. This suggests these AEs primarily act as clinical manifestations of underlying frailty, a surrogate indicator for mortality risk. Furthermore, our findings suggest that these AEs and poor long‐term prognosis are primarily driven by systemic factors (PS and CCI). These outcomes imply that pre‐procedural frailty leads to acute systemic complications, which in turn reflect long‐term mortality risk. This integrated perspective suggests that PS and CCI, and not endoscopic or oncological factors, could serve not just as general predictors of survival, but as useful indices for determining treatment indications.

Delayed bleeding, a major procedure‐related AE, is influenced more by systemic comorbidities, such as antithrombotic therapy and dialysis, than by lesion‐related factors. Consistent with previous multivariate analyses [12, 13], age was not significantly associated with delayed bleeding in our study.

Although pneumonia has been frequently reported in elderly patients [2, 4, 5], other non‐procedure‐related AEs that warrant particular attention in this population, such as those with severe comorbidities or compromised general conditions, have rarely been described. Regarding surgical treatments that are more invasive than ESD, a meta‐analysis found no age‐related increase in procedure‐related AEs [14]. However, elderly patients experience a higher incidence of postoperative AEs, such as in‐hospital mortality and respiratory or cardiovascular events. Our findings indicated that procedure‐related AEs did not occur more frequently in elderly patients, whereas non‐procedure‐related AEs, such as pneumonia, were more common. According to Ueda et al., factors such as physical status, nutritional status, and medical history are significantly associated with severe postoperative AEs in elderly patients undergoing surgery [15]. In the present study, non‐procedure‐related AEs were associated with PS ≥ 2 and CCI ≥ 2 among elderly patients. Notably, PS ≥ 2 was an independent risk factor, which may indicate its predictive role for these AEs within the limitations of our cohort size.

Thromboembolic events, such as cerebrovascular events, can occur during the withdrawal period of antithrombotic agents and can result in critical situations, including fatal clinical outcomes. The incidence of thromboembolic events in the perioperative setting among patients receiving anticoagulant therapy has been reported in several studies to range from 0.2% to 0.4% [16, 17, 18]. In the present study, two thromboembolic events, one case of cerebral infarction and one case of myocardial infarction, occurred in patients aged ≥80 years. The former developed after delayed bleeding and occurred during a three‐day interruption of anticoagulant therapy; this event was ultimately fatal. The latter occurred despite only a one‐day interruption of antiplatelet therapy on the day of treatment. An increased incidence of thromboembolic events during the temporary interruption of anticoagulant therapy has been reported in elderly patients receiving edoxaban, a direct oral anticoagulant [19], and these findings suggest that caution should be exercised during the interruption of antithrombotic therapy in elderly patients because of the risk of thromboembolic events.

Although the overall procedure‐related mortality rate in our study was low (0.2% [two cases]), both fatalities occurred in elderly patients with poor PS or high CCI. One of these patients, as mentioned earlier when discussing thrombotic events, had a poor PS (PS ≥ 2) and died of pneumonia after a cerebral infarction. The other patient had an underlying hematological disease and liver cirrhosis, resulting in a high CCI (CCI ≥ 2). Although deep submucosal invasion was suspected, ESD was performed to achieve local control. However, this resulted in a non‐curative resection (eCuraC‐2). The patient experienced repeated delayed bleeding, and the general condition rapidly deteriorated, leading to death. Despite the small sample size, these findings align with those in a previous report, indicating that multiple comorbidities increase the risk of short‐term mortality after ESD in the elderly [20].

Consistent with previous literature, established indicators of mortality, including the albumin level, CCI, PS, and age, independently predicted poorer OS in elderly patients [21, 22]. Consistent with previous studies, non‐curative patients were not identified as significant prognostic factors [21]. Moreover, our analysis included patients with non‐cancerous lesions, and there was no significant difference in prognosis compared with patients with cancerous lesions. These results collectively indicate that among patients undergoing gastric ESD, oncological factors have a negligible impact on long‐term survival; instead, comorbidities and systemic health status should be interpreted as indicators of a baseline life expectancy. Notably, our study identified short‐term non‐procedure‐related AEs as a significantly poor prognostic factor for OS. To the best of our knowledge, this association has not been reported previously. Most of these AEs were manageable (CTCAE grade ≤3), and we interpret their presence not as the primary cause of death, but as a clinical manifestation that unmasks underlying frailty. Thus, these AEs are a surrogate indicator for poor long‐term survival. This novel finding further underscores the increased importance of pre‐procedural assessments for short‐ and long‐term prognoses.

Yamada et al. demonstrated that in elderly patients aged ≥85 years with clinical T1N0 EGC, ESD was associated with superior OS compared with no treatment [23]. Given the potential for tumor progression to cause bleeding, resection is generally recommended to improve the prognosis when the procedural risk is low. A balanced consideration of the therapeutic benefits and procedural risks of systemic manifestations of frailty is essential when determining the indications for gastric ESD in vulnerable patients. Consequently, CCI and PS, which were significantly associated with non‐procedure‐related AEs and OS in this study, emerge as valuable clinical indices for determining treatment indications.

This study had some limitations. First, its retrospective nature introduced biases related to data collection and confounding factors. Although the elderly patients had a slightly higher proportion of gastric cancer and a slightly larger lesion size, these lesion‐related factors were not identified as predictors of AEs in the analyses. Therefore, their impact as sources of selection bias is likely to be minimal. Second, the management of antithrombotic agents was not prospectively standardized, and the timing and extent of withdrawal were left to the discretion of individual physicians, which is generally in accordance with the current guidelines. Efforts have also been made to minimize the duration of drug interruption. Third, until approximately 2020, both institutions used a 9–10‐day clinical pathway for gastric ESD, which was later shortened to at least seven days, making a direct comparison of hospital stays between periods difficult. Despite these limitations, our large two‐institution cohort suggests high generalizability.

In conclusion, among elderly patients aged ≥80 years, the non‐procedure‐related AE rate was significantly higher than that in non‐elderly patients. Moreover, these AEs have been identified as a significant poor prognostic factor for OS. Notably, PS ≥ 2 and CCI ≥ 2 serve as critical markers that are associated with non‐procedure‐related AEs and long‐term prognosis. These findings highlight the importance of comprehensive pre‐operative assessment focusing on systemic health status, a critical index for determining treatment indications in elderly patients aged ≥80 years undergoing gastric ESD, rather than chronological age alone.

## Author Contributions

**Conceptualization and formal analysis**: H.O. **Investigation**: H.O., T.I., H.O, K.H., N.T., H.F., H.S., T.A., H.S., N.F., K.F., S.K., H.I., S.T., and M.O. **Methodology**: H.O., Y.M., and T.A. **Drafting of the article**: H.O. **Writing – original draft preparation**: H.O. **Writing – review & editing**: H.O., Y.M., T.A., and H.I. **Supervision**: H.O.

## Conflicts of Interest

The authors declare no conflicts of interest.

## Funding

The authors have nothing to report.

## Consent

The opt‐out was posted on the website.

## Ethics Statement

The study protocol was approved by the Institutional Review Boards of Ikeda Municipal Hospital (registration number: 3531) and Itami City Hospital (registration number: 2715).

## Supporting information




**FIGURE S1**: Receiver operating characteristic (ROC) curve of the Charlson Comorbidity Index (CCI) for predicting non‐procedure‐related adverse events. The area under the curve (AUC) is 0.72 (95% confidence interval [CI], 0.621–0.818). Based on this analysis, the optimal cutoff value for the CCI was determined to be 2.

**FIGURE S2**: Kaplan–Meier curves showing overall survival in elderly (≥80) and non‐elderly patients (<80). Overall survival was significantly poorer in elderly patients (dashed line) than in non‐elderly patients (solid line) (log‐rank test, *p* < 0.001). The number at risk for each time point is shown in the following graph.

**TABLE S1**: Univariate analysis of factors associated with adverse events in elderly patients.
**TABLE S2**: Multivariate logistic regression analysis of factors associated with adverse events in elderly patients.
