# Supplementary figures and images for: Short‐ and Long‐Term Outcomes of Endoscopic Submucosal Dissection for Gastric Lesions in Elderly Patients Aged 80 Years or Older: Focus on Non‐Procedure‐Related Adverse Events
Source: DEN Open. 2026 Apr 19;6(1):e70334. doi: 10.1002/deo2.70334 (PMC13092489; doi:10.1002/deo2.70334)

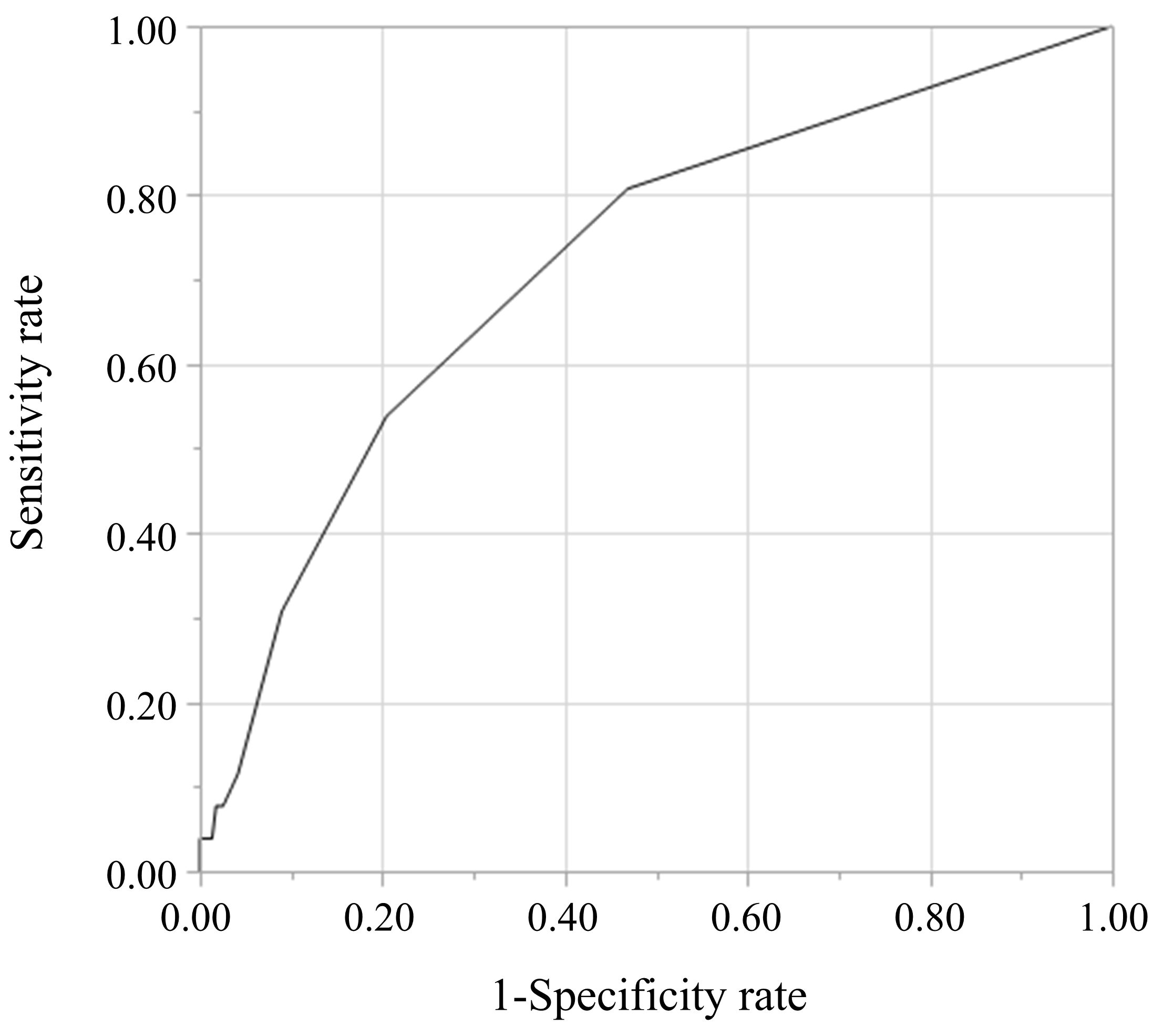

Supplement: Supplementary file 1 — FIGURE S1: Receiver operating characteristic (ROC) curve of the Charlson Comorbidity Index (CCI) for predicting non‐procedure‐related adverse events. The area under the curve (AUC) is 0.72 (95% confidence interval [CI], 0.621–0.818). Based on this analysis, the optimal cutoff value for the CCI was determined to be 2. [file DEO2-6-e70334-s002.tif]

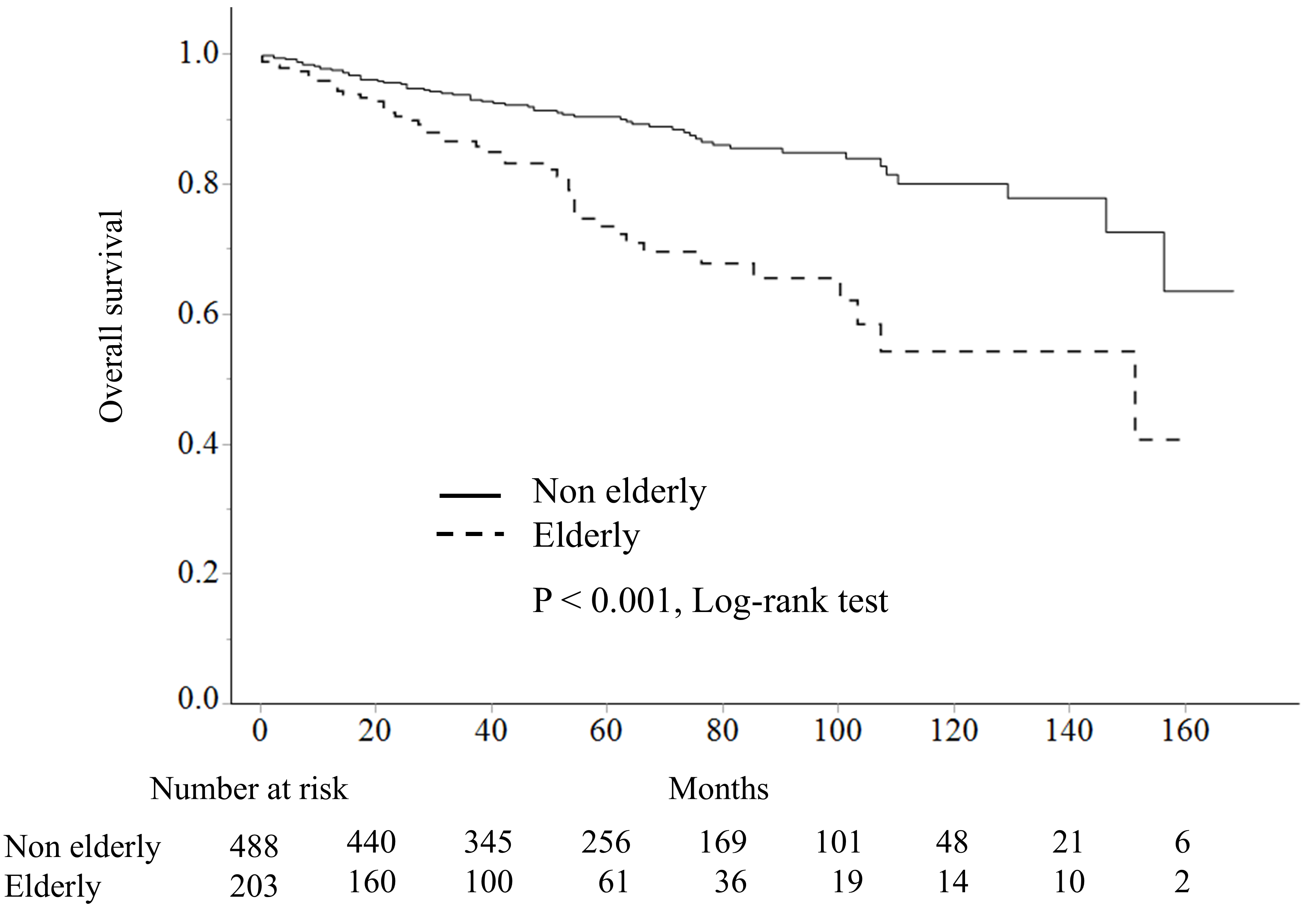

Supplement: Supplementary file 2 — FIGURE S2: Kaplan–Meier curves showing overall survival in elderly (≥80) and non‐elderly patients (<80). Overall survival was significantly poorer in elderly patients (dashed line) than in non‐elderly patients (solid line) (log‐rank test, p < 0.001). The number at risk for each time point is shown in the following graph. [file DEO2-6-e70334-s003.tif]
