# Supplementary material for: Short‐ and Long‐Term Outcomes of Endoscopic Submucosal Dissection for Gastric Lesions in Elderly Patients Aged 80 Years or Older: Focus on Non‐Procedure‐Related Adverse Events
Source: DEN Open. 2026 Apr 19;6(1):e70334. doi: 10.1002/deo2.70334 (PMC13092489; doi:10.1002/deo2.70334)
Supplement: Supplementary file 3 — TABLE S1: Univariate analysis of factors associated with adverse events in elderly patients. TABLE S2: Multivariate logistic regression analysis of factors associated with adverse events in elderly patients. [file DEO2-6-e70334-s001.docx]

Supplementary Table 1. Univariate analysis of factors associated with adverse events (AE) in elderly patients

|  | AE  (n = 40) | Non-AE  (n = 251) | P-value |
| --- | --- | --- | --- |
| Very elderly ≥85, n (%) | 15 (37.5) | 25 (30.7) | 0.389 |
| Sex (male), n (%) | 29 (72.5) | 168 (66.9) | 0.484 |
| Antiplatelet therapy | 18 (45.0) | 78 (31.1) | 0.082 |
| Anticoagulant therapy | 5 (12.5) | 27 (10.8) | 0.744 |
| Hemoglobin (g/dL, mean ± SD) | 12.6 ± 1.8 | 12.6 ± 1.7 | 0.874 |
| Albumin (g/dL, mean ± SD) | 3.77 ± 0.54 | 3.90 ± 0.40 | 0.077 |
| eGFR (mL/min/1.73 m^2^, mean ± SD) | 53.3 ± 16.2 | 59.1 ± 17.8 | 0.055 |
| BMI (kg/m^2^, mean ± SD) | 22.4 ± 3.4 | 23.0 ± 3.3 | 0.366 |
| CCI ≥2 | 20 (50.0) | 65 (25.9) | 0.002 |
| PS ≥2 | 10 (25.0) | 16 (6.4) | <0.001 |
| Location (U/M/L/remnant), n | 5 / 11 / 21 / 3 | 34 / 82 / 124 / 11 | 0.782 |
| Macroscopic type (elevated/flat or depressed), n | 17 / 23 | 145 / 106 | 0.071 |
| Lesion size (mm, mean ± SD) | 17.9 ± 7.8 | 18.0 ± 12.4 | 0.991 |
| Procedure time (min, mean ± SD) | 91.8 ± 60.9 | 76.6 ± 59.2 | 0.139 |
| Operator experience (trainee) †, n (%) | 17 (42.5) | 103 (41.0) | 0.861 |
| Curability‡ eCuraC-2/nonC-2/non-resected, n | 6 / 32 / 2 | 31/ 217 / 3 | 0.195 |

SD, standard deviation; eGFR, estimated glomerular filtration rate; BMI, body mass index; CCI, Charlson Comorbidity Index; PS, Eastern Cooperative Oncology Group performance status

†Trainee endoscopists were defined as those who had performed ≤30 gastric ESD procedures.

‡Curability was evaluated using a scoring system^11^. Non-resected cases refer to lesions for which ESD could not be successfully completed. NonC-2 lesions exclude eCuraC-2 and non-resected cases, including non-cancerous lesions.

Supplementary Table 2. Multivariate logistic regression analysis of factors associated with adverse events in elderly patients

|  | Odds ratio | 95% (CI) | P-value |
| --- | --- | --- | --- |
| CCI ≥2 | 2.38 | 1.16 – 4.83 | 0.018 |
| PS ≥2 | 3.87 | 1.52 – 9.49 | 0.005 |

CI, confidence interval; CCI, Charlson Comorbidity Index; PS, Eastern Cooperative Oncology Group performance status
